# Surgical management of eumycetoma: experience from Gezira Mycetoma Center, Sudan

**DOI:** 10.1186/s41182-018-0129-2

**Published:** 2019-01-14

**Authors:** Mohamed D. A. Gismalla, Gamal M. A. Ahmed, Mogahid M. MohamedAli, Sami M. Taha, Thouria A. Mohamed, Ahmed E. Ahmed, Lamia S. Hamed

**Affiliations:** 1 Department of surgery, Faculty of Surgery, Gezira University, Wad Medani, Gezira Sudan; 2 Gezira Mycetoma Center, Ministry of Health, Wad Medani, Gezira Sudan

**Keywords:** Amputations, Eumycetoma, Mycetoma, Sudan, Surgical excision, Surgery

## Abstract

**Background:**

In this study, we share our experience of different operative techniques undertaken on 584 eumycetoma patients in the Gezira Mycetoma Center.

**Methods:**

This is a retrospective, descriptive, hospital-based study, conducted to review the surgical treatment of eumycetoma patients. We included all patients diagnosed with eumycetoma who underwent a surgical operation in the center during January 2013–December 2016.

**Results:**

A total number of 1654 patients were seen during the study period, and their records were revised, while 584 (35.3%) of them underwent an operation and included in the study. There was a male predominance 446 (76.4%). Surgical excision of mycetoma was the commonest operation performed among 513 (87.8%) patients in comparison with amputation 71 (12.2%). Below-knee amputation and toe amputation are the commonest types of amputation in 36 (6.1%) and 14 (2.3%) patients, respectively. Clinical features determining the type of operation performed included the size of the lesion, whether or not a bone was involved, and the feasibility of primary closure. A wide surgical excision (WSE) is performed mainly when the bone is not involved and when moderate or primary closure is possible or reconstruction is feasible. Amputations will typically follow identifying bone involvement, secondary infection, and an already disabled patient.

**Conclusion:**

The commonest procedure in our series was WSE and primary skin closure undertaken when the lesion was small (< 5 cm); there was no bone involvement, and the skin closure was achievable. Larger lesions (> 10 cm) without bone involvement were treated with excision and flap/graft. Bone involvement and large primary lesions were more likely to be managed by amputation. Recurrent and relapse of mycetoma were observed in patients with bone involvements or presented with recurrent mycetoma for the second time.

## Background

Recently, mycetoma was recognized by the World Health Organization as a neglected tropical disease. It is defined as a chronic skin and subcutaneous swelling caused either by eumycetoma or actinomycetoma [1]. It has a worldwide distribution but is endemic in tropical and subtropical countries across the “mycetoma belt.” This runs from India to Yemen and goes through Sudan, Senegal, and onto South America and Mexico [2]. In Sudan, mycetoma is common in Gadarif, Kassala, Sinnar, Gezira, Khartoum, Kordofan, and Darfur states. Gezira state was recognized as having one of the highest incidences of mycetoma. Fahal et al. conducted a review of their experience from the Mycetoma Research Center from 1991 to 2014. They reported that of the 2476 patients attending the center, 792 (31.9%) were from Gezira state [3].

Management of mycetoma is determined mainly by the causative organism infections. Actinomycetoma responds well to medical treatments like Septrin and vancomycin. Surgery has a limited role and is mainly used for unresponsive cases or combined with medical treatments [4]. In contrast, eumycetoma has a poor response to medical therapy (anti-fungal such as azoles). Recently, itraconazole has been used in doses of 100–200 mg per day for 6–12 months with/or without combination with surgical management [5]. In the absence of effective medical treatment for eumycetoma, surgical approaches are all that is available. The commonest surgical option is wide surgical excision which is recommended for small well-circumscribed lesions (≥ 5 cm) [6]. The other options are wide surgical excision plus flap can be done for big lesions when primary closure is not applicable and amputation can be done for toes, fingers, or big complicated limbs (hand or foot) [7].

Classification of the causative organism is undertaken by microscopic examination of the grains or using fine-needle aspirations cytology. This will show the non-caseating granulomatous inflammatory feature [8]. Once eumycetoma is established as the cause, the second most important step is to determine the local extension of the disease and evaluation of bone status by using radiological techniques [9]. In spite of bridging the gap of knowledge by mycetoma group, still the management’s outcome is not promising; there is a lack of detailed indications, types, and surgical techniques toward mycetoma [10]. The treatment depends mainly on physician preference and experience. These raise the need for global or regional guidelines.

Gezira Mycetoma Center was established in 2012 after Memorandum Corporation between Blue Nile National Institute for Communicable Disease, Gezira University, and Gezira State Ministry of Health, Sudan Federal Ministry of Health, Sudan Ministry of Social Welfare and Health Insurance Corporation [11]. It covers the Gezira state and other nearby states. A total number of 60–80 patients are seen per month in the referred clinic with 20 to 30 operations undertaken each week. In this study, we share our experience of the different operative techniques used to treat our eumycetoma patients, looking at the factors that determine the choice of operation, at Gezira Mycetoma Center.

## Methods

### Study design

This is a retrospective, descriptive, hospital-based study, conducted during January 2013–December 2016 to review the surgical treatment of eumycetoma patients. All patients’ records were reviewed during the study period.

### Inclusion criteria

We included all patients who are diagnosed with eumycetoma (fungal) and underwent surgical treatment in the center during the study period.

### Exclusion criteria

Any patient operated in other hospitals, has unresectable eumycetoma lesions, or has actinomycetoma was excluded.

### Diagnostic evaluation

Clinical assessment is undertaken for all patients presenting to the center. These evaluations determine the history of the lesion, duration, presence, and color of the grain as well as a local and regional examination of the limp or mass. Then, imaging radiological study is performed to patients to evaluate the bone status and soft tissue swelling.

Diagnosis of eumycetoma or actinomycetoma includes fine-needle aspiration cytology and histological diagnosis and clinical evaluation (discharging grain or sinuses). A histological examination can be done by using hematoxylin and eosin (H&E) stain which shows the non-caseating granulomatous necrosis. If the result is not conclusive, other stains can be used like periodic acid-Schiff and methylamine silver stains. The final result in our clinical work cannot define the subspecies for each group.

### Operative techniques

Preoperative clinical evaluation, routine urine samples, blood test, and X-rays were performed according to each patient’s condition. The choice of anesthesia was typically spinal or general anesthesia according to the lesion site or age of the patients. For the upper or lower limb lesion, tourniquet had been used almost all the time while in cases of a trunk mycetoma, diathermy has been used. Careful surgical dissection was performed to excise the lesion completely, followed by washing and irrigation of surgical site with normal saline and iodine solution. Amputation of the toes, fingers, forearm, or leg was taken only after counseling and discussion with the patient.

The hospital stay was 2–3 days. Outpatient reviews were at 2 weeks, 1 month, and 3 months till 6 months, 12 months, and 18 months. Wound healing, deformity, and recurrence were checked during these periods. Itraconazole tablets 200–400 mg (anti-fungal agents) were given routinely perioperatively according to Suleiman et al.’s guideline (Mycetoma Research Center, Khartoum) [6]. For small-sized mycetoma ≤ 5 cm, we give itraconazole tablets only postoperatively for 3 months while for moderate- and big-sized lesion ≥ 5 cm, patient receives the treatments for 6 months before and after the operation.

### Data collection and analysis

Flowchart sheets were used to collect the data. Age, sex, residence, and occupation were checked. Information regarding the presence of sinus (grains), recurrence mycetoma, and X-ray evaluations was determined. Types of operation done and factors to select each operation were determined.

## Results

Patients’ characteristics were shown in Table 1. A total number of patients who were included in this study were 584. The median range of follow-up was 18 months (range between 12 and 24 months). The total number of patients who are less than 20 years of age is 92 (14.6%). The male patients are predominant with 446 (76.4%). Most of the patients in this study reside in Gezira state (472 (80.8%)). Farmers (214 (36.7%)) and (182 (31.2%)) students were the commonest jobs among patients in the study. Patients who had recurrent mycetoma and recruited in this study are 94 (16.1%). Discharging grains and sinus were found in 461 patients (78.9%). Radiological evaluations revealed that bone was not involved in 483 (82.7%) patients.

**Table 1:** Characteristics of patients in the study (*N* = 584)

Variables		Frequency	Percent
Age distribution	< 10	29	4.9
	10–19	63	10.7
	20–40	279	47.7
	> 40	213	36.7
Sex	Male	446	76.4
	Female	138	23.6
Residence state	Gezira State	472	80.8
	Sinnar State	42	7.2
	Gadarif State	24	4.2
	Kassala State	15	2.5
	Others	31	5.3
Occupation	Farmer	214	36.7
	Animal breeder	84	14.4
	Housewife	103	17.7
	Student	182	31.2
Recurrent mycetoma	Yes	94	16.1
	No	490	83.9

Surgical excision of mycetoma was the commonest operation done in the center among 513 (87.8%) patients in comparison with amputation which performed between 71 (12.2%) patients (Table 2). Wide surgical excision and primary closure was the commonest surgical operation, and it was done in 480 (82.4%) patients. Below-knee amputation and toe amputation are the commonest types of amputation in 36 (6.2%) and 14 (2.4%) patients, respectively.

**Table 2:** Factors associated with types of surgical operation (*N* = 584)

		Total	Wide surgical excision	WSE graft or flap	Amputation	*p* value
Size	Small, 5 cm	491	472	0	19	< 0.01
	Moderate, 5–10 cm	35	7	21	7	
	Big, more than 10 cm	35	2	11	45	
X-ray	Bone involve	101	0	30	71	< 0.01
	Bone not involve	483	481	2	0	
Sinus	Yes	461	358	32	71	< 0.01
	No	123	123	0	0	
Total		584	481	32	71	

**Fig. 1:**
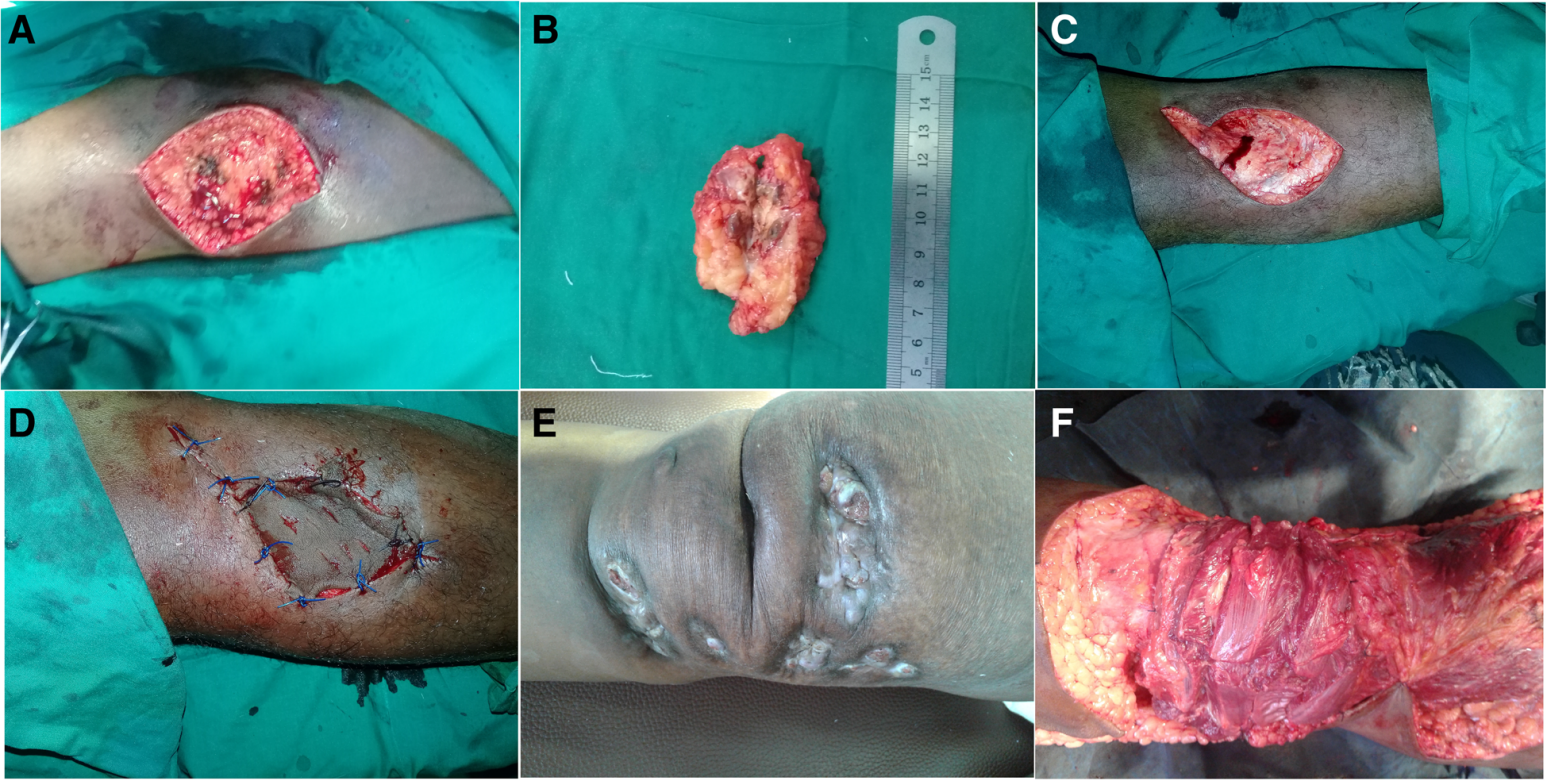
Wide surgical excision (WSE) and primary closure, skin graft, or flap. **a** WSE with a margin of mycetoma lesion. **b** The excised same lesion which measures about 6 cm. **c**, **d** WSE with margin defect closed with skin graft. **e** Recurrent knee mycetoma. **f** Reconstruction after excision with gastrocnemius flap

**Fig. 2:**
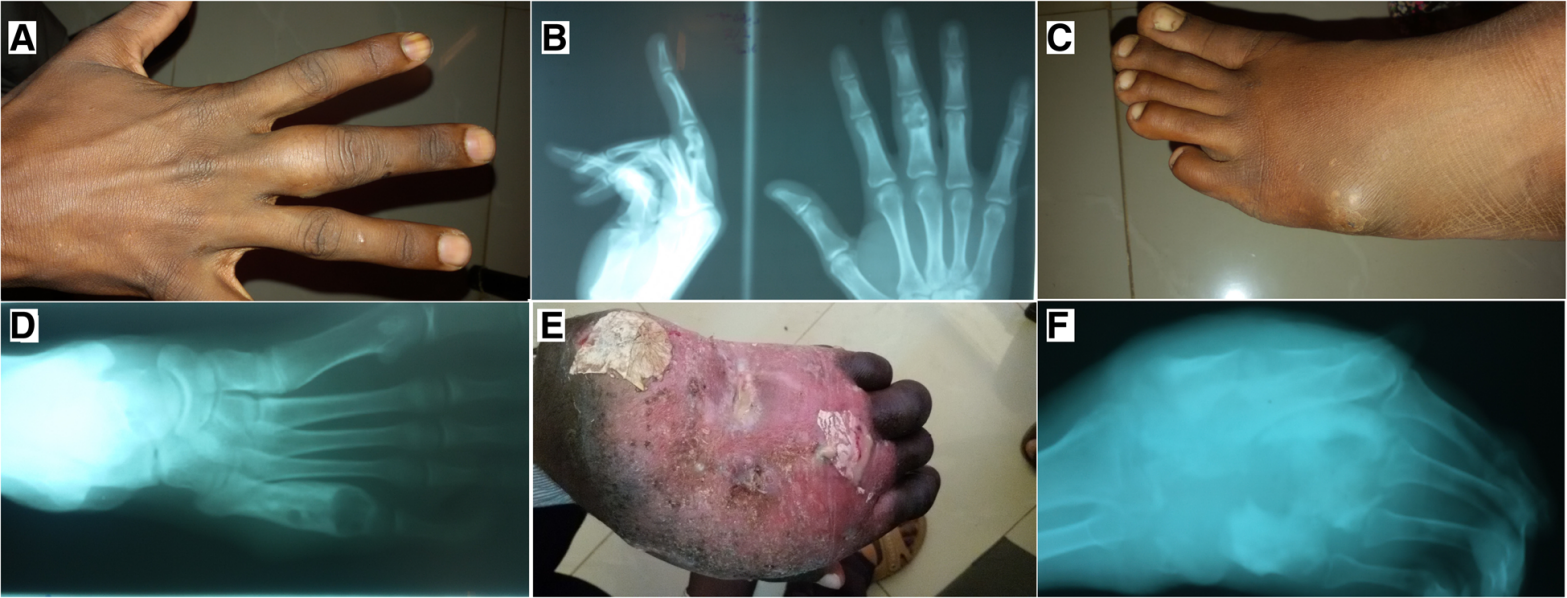
Pre-amputation images of the finger, toe, and hand with X-ray showing mycetoma. **a** Right middle finger eumycetoma (proximal phalanx). **b** PA and lateral X-ray view of the same patients showing cavity and evidence of mycetoma (this patient treated by finger Ray’s amputation). **c** Left toe mycetoma with sinus cavity. **d** X-ray was showing cavity and mycetoma (this patient treated by toe Ray’s amputation). **e** Hand showing mycetoma deformity, discoloration, and loss of function. **f** X-ray of the same patient was showing a deformity, cavitation, and mycetoma (this patient treated by below-elbow amputation)

There were several factors which are believed to determine the type of surgical operation in our series. Wide surgical excision and primary closure depended on the size of the lesion, bone not involved, and feasibility of primary closure (Fig. 1; Table 2). Wide surgical excision (WSE) plus skin graft or flap (Fig. 1) depends on bone involvements, moderate or big size, and feasibility of reconstruction. Factors which determine the selection of amputation are bone involvement, secondary infection, disability of patients, and patients request options (Fig. 2; Table 3).

**Table 3:** Factors associated with types of amputations (*N* = 71)

		Total	BKA	AKA	Elbow amputation	Syme’s amputation	Ray’s amputation
Secondary infection	Yes	25	13	3	3	5	1
	No	46	23	1	0	4	15
Cannot walk (disability)	Yes	52	36	4	3	9	0
	No	19	0	0	0	0	19
Patient request	Yes	19	0	0	0	0	19
	No	52	36	4	3	9	0

*BKA* below-knee amputation, *AKA* above-knee amputation

There is a significant statistical relation between the size of the tumor and bone involvements and selection of patients. After an average period of follow-up (18 months), the recurrence rate among those patients is 32 (5.5%). There is a significant relation between recurrent mycetoma in our patients in this study and bony involvement, recurrence mycetoma, types of operation, and size of the lesion shown in Table 4.

**Table 4:** Factors associated with recurrent mycetoma (*N* = 584)

	Variables	Recurrence after surgery		Total	*p* value
		Yes	No		
Bone involvements	Yes	12	89	101	< 0.01
	No	20	463	483	
First recurrence mycetoma^a^	Yes	16	78	94	< 0.01
	No	16	474	490	
Operation	Wide surgical excision	18	463	481	< 0.01
	WSE graft or flap	8	24	32	
	Amputation	6	65	71	
Size	Small, 5 cm	17	474	491	< 0.01
	Moderate, 5–10 cm	7	28	35	
	Big, more than 10 cm	8	50	58	

^a^Patient presented to the center with already recurrent mycetoma

## Discussion

Most of the patients in this study underwent wide surgical excision and primary closure. Surgical excision is the treatment of choice for treating eumycetoma if it is achievable. The characters these patients most likely to do well here are those with small lesions and without bone involvements [12]. Surgical excision needs a careful surgical dissection in a bloodless field and an experienced hand to reduce the recurrence [6]. Surgical excision and reconstruction by flap and/or graft can be done for moderate or large lesions when bone is not involved and the primary closure is not feasible [7].

Eumycetoma is a disfiguring and disabling disease. It is the third leading cause of amputation in Sudan after diabetic-related problems (infection and vascular) and trauma [13]. Although amputation is avoided as much as possible in Sudan, it is indicated among mycetoma patients in about 25–50% of cases. The published rate of amputations for mycetoma worldwide ranges from 25 to 50% [13, 14]. In this study, the commonest amputations are below-knee and toe amputations. Limb amputation is performed for huge eumycetoma (≥ 10 cm), with a secondary bacterial infection, to save patient life [14]. In our series, amputation of toes and fingers were undertaken to achieve cure, when bone was involved and WSE not possible. Limb amputation was done for disfiguring lesions, secondary infection, and patients already unable to walk.

Bone status should be determined in any mycetoma patients especially eumycetoma. The radiological study is cheap, available, and easy to interpret. In Sudan, Fahal et al. [15] reported bone involvement among 73% of mycetoma patients; the radiological abnormality varies from soft tissue mass and bone destruction to periosteal reactions. Any patient without bone involvements has a better chance to be cured. In this study, we depended mainly on radiological (X-ray) evaluation of bone. Then, patients are categorized into two main groups (bone involvements or not involved). According to the bone status, we can judge the types of operation that can be offered to the patient. If a bone is not involved wide surgical excision can be chosen or amputations for bone involvements.

In this study, the commonest operation for eumycetoma is wide surgical excision and primary skin closure. This operation was selected due to the small-sized lesion (less than 5 cm), no bone involvements, and the skin can be closed easily. Any big-sized lesion without bone involvements can be treated with excision and flap/graft. Bone involvements with massive mycetoma are predicting factors for amputation.

## Conclusion

This study highlights the factors which determine surgical management choices for eumycetoma in Sudan. Which operation is chosen mainly depends on the size of the lesion and bone involvements. Amputations were performed among patients with huge lesions, disabled, and requested amputation for cure. One of the limitations of this study is that we did not analyze the other factors which can affect the outcome like time of presentation, preoperative medication, and subspecies of mycetoma.

## Abbreviations

WSE: Wide surgical excision
